# Psychosocial Determinants of Mental Health in Older Adults Living With HIV: The Protective Role of Resilience Against Internalized Stigma

**DOI:** 10.1155/jare/5024899

**Published:** 2026-09-25

**Authors:** Victoria Tischer Sawka, Guilherme Welter Wendt, Angelo Brandelli Costa

**Affiliations:** ^1^ Faculty of Medicine, Western Paraná State University, Francisco Beltrão, Brazil; ^2^ Department of Psychology, John Cabot University, Rome, Italy; ^3^ Department of Psychology, Pontifical Catholic University of Rio Grande do Sul, Porto Alegre, Brazil, pucrs.br

**Keywords:** anxiety, Brazil, depression, HIV, internalized stigma, network analysis, older adults, path analysis, resilience, stigma index 2.0

## Abstract

Despite comprising nearly one in 10 people living with HIV (PLHIV) in Brazil, older adults aged 60 years and above remain profoundly underrepresented in psychosocial research. This multicenter study is the first age‐restricted (≥ 60 years) subgroup analysis of the Brazilian PLHIV Stigma Index 2.0. The present secondary analysis yielded a sample of 138 older adults (M age = 64.3 years, SD = 4.1; 52.9% male; 57.2% Black or Brown). Path analysis with 5000‐resample bootstrapping and full‐information maximum likelihood estimation confirmed a statistically significant indirect effect of general stigma on depression mediated by internalized stigma (indirect effect = 0.087, 95% CI [0.037, 0.173], *p* = 0.009), with no significant direct pathway (c’ = 0.058, *p* = 0.324). A parallel model confirmed that internalized stigma also mediated the effect of general stigma on anxiety (indirect effect = 0.071, 95% CI [0.029, 0.146], *p* = 0.012). A moderated mediation model testing whether resilience buffers the stigma–internalization pathway found that resilience did not significantly moderate the a‐path (interaction *b* = 0.003, *p* = 0.817) but maintained a significant inverse main effect on internalized stigma (*b* = −0.091, *p* = 0.014), supporting an upstream rather than buffering mechanism. Complementary network analysis using regularized partial correlations (EBICglasso) confirmed internalized stigma as the dominant psychosocial hub (strength centrality = 0.896). Network predictability was highest for depression (*R* ^2^ = 0.41) and internalized stigma (R^2^ = 0.32). Neither age nor time since diagnosis independently predicted psychological distress. These findings support a mechanistic model in which internalized stigma mediates the effect of social discrimination on both depressive and anxious symptomatology, and resilience acts as an upstream protective factor. Stigma‐focused, resilience‐building, and aging‐sensitive psychosocial interventions are indicated as priority components of HIV care for aging populations.

## 1. Introduction

Advances in antiretroviral therapy (ART) have fundamentally transformed HIV from a terminal illness into a manageable chronic condition, enabling prolonged survival and near‐normal life expectancy for people who have uninterrupted access to treatment. A direct epidemiological consequence of this success is the progressive aging of the global people living with HIV (PLHIV) population: as of 2024, approximately 40.8 million people worldwide were living with HIV, with a growing proportion aged 50 years and older [1]. In Brazil, an estimated 1 million PLHIV/AIDS, and the proportion aged 50 or older has risen steadily, reflecting the same demographic shift observed globally [2]. Yet despite their increasing numerical significance, older adults living with HIV are dramatically underrepresented in psychosocial research. The dominant intervention literature focuses on transmission, adherence, and younger populations, leaving a critical evidentiary gap precisely where the clinical burden is shifting [3].

HIV‐related stigma operates across multiple, partially separable dimensions. Perceived or enacted stigma encompasses external experiences of discrimination, social rejection, and prejudice directed toward PLHIV by others. Internalized stigma, by contrast, refers to the process by which individuals incorporate those external judgments into their own self‐concept, resulting in shame, guilt, and diminished self‐worth [4, 5]. Anticipated stigma—concern about future stigmatizing encounters—has been shown to exert independent effects on health outcomes distinct from both experienced and internalized stigma [6, 7], although it was not measured in the present study. All three dimensions are independently associated with adverse mental health outcomes, but internalized stigma is increasingly recognized as the more proximal determinant of psychological distress, because it operates from within the self‐regulatory system rather than as an external event [8]. For older adults, this burden is compounded by ageism: the intersection of HIV‐related stigma with age‐based discrimination creates a context of double stigmatization, amplifying psychological vulnerability beyond what either stigma source would produce independently [3, 9].

Resilience—understood as the dynamic capacity for positive adaptation in the face of adversity—has attracted increasing attention as a potential protective factor in this population. The present study uses the PLHIV Resilience Scale [10], a measure developed and validated specifically for PLHIV that captures HIV‐specific coping, empowerment, and community connection, rather than general trait resilience. This distinction is important because HIV‐specific resilience may tap processes—such as stigma‐resistance skills and peer solidarity—that are more directly actionable in clinical interventions than generic coping capacity. Two alternative mechanistic pathways are theoretically possible: resilience may operate directly on emotional regulation to reduce depression and anxiety independently of stigma experiences, or it may operate indirectly by attenuating the internalization of external discrimination [11, 12]. Turan et al. [6] demonstrated in a sample of 1405 women living with HIV that dispositional resilience moderated the effect of experienced stigma on depression (*B* = −1.94, *p* = 0.007) but did not moderate the effect of anticipated stigma, suggesting that the buffering role of resilience may be specific to certain stigma types. Discriminating between these pathways is not merely an academic question: it determines whether psychosocial interventions should primarily target general coping capacity or specifically address stigma‐internalization processes.

Network analysis offers a complementary analytical lens that does not require a priori specification of directional hypotheses. By mapping the associational structure among psychosocial variables and estimating each variable’s centrality within the system, network methods identify potential strategic intervention targets—the nodes whose perturbation would most broadly affect system functioning [13, 14]. The identification of a central hub within a psychosocial symptom network suggests that interventions targeting that hub would simultaneously influence multiple connected outcomes, conferring efficiency advantages over single‐symptom approaches. Network and path analysis approaches are complementary: the former is exploratory and identifies the overall structure and central hubs without directional assumptions, while the latter is confirmatory and tests specific theoretically motivated directional pathways. Presenting both in sequence allows the reader to first appreciate the full associational landscape and then focus on the specific mediation pathway of interest.

The present study addressed these gaps by employing both structural equation modeling and network analysis to examine associations among internalized stigma, general stigma, resilience, anxiety, depression, age, and time since diagnosis in a multicenter community‐based sample of older adults living with HIV across Brazil. This is the first analysis to restrict the Stigma Index Brazil 2.0 sample to adults aged 60 years and above, enabling age‐specific inferences that are not possible in the full mixed‐age sample [15]. We hypothesized that (1) internalized stigma would mediate the relationship between general stigma and both depression and anxiety; (2) resilience would predict reduced internalized stigma but would not significantly moderate the stigma–internalization pathway, operating instead as an upstream protective factor; and (3) network analysis would identify internalized stigma as the central hub of the psychosocial system, consistent with its role as the proximal mediating variable. These hypotheses were grounded in identity‐threat models of stigma [5], meta‐analytic evidence linking internalized stigma to depression across diverse PLHIV samples [16], and prior evidence that resilience is inversely associated with internalized stigma in older PLHIV [17].

## 2. Method

### 2.1. Design and Setting

This was a quantitative, cross‐sectional study based on secondary analysis of data from the People Living with HIV Stigma Index 2.0 project (PLHIV Stigma Index 2.0; [18]), an internationally validated, community‐led data collection initiative administered across seven Brazilian state capitals (São Paulo, Rio de Janeiro, Belo Horizonte, Recife, Salvador, Manaus, and Porto Alegre). The parent study recruited 1784 PLHIV of all ages (M age = 40.14, SD = 12.95) using a community‐based participatory approach in which trained PLHIV interviewers administered the survey on tablet computers to purposively sampled respondents recruited through PLHIV networks, community‐based organizations, HIV clinics, and snowball sampling [15]. Eligibility criteria for the parent study included being aged 18 years or older, having known one’s HIV‐positive status for at least one year, and residing in the study area. Data collection was conducted between 2018 and 2019. The multicenter design, spanning geographically and socioculturally distinct urban settings, strengthens the representativeness of the sample relative to studies conducted in single sites, and partially mitigates the generalizability limitations inherent in community convenience sampling within any one city.

### 2.2. Participants

For the present secondary analysis, the parent sample was restricted to individuals aged 60 years or older—the age threshold used by the World Health Organization and Brazilian public health policy to define older adults—yielding a subsample of 138 participants. Of these, 52.9% were male (*n* = 73) and 47.1% were female (*n* = 65); 57.2% identified as Black or Brown. Mean age was 64.3 years (SD = 4.1; range: 60–76). Mean time since HIV diagnosis was 17.65 years (SD = 9.32). The overwhelming majority (97.1%, 134/138) were receiving ART, a clinical characteristic that renders this sample broadly representative of the contemporary Brazilian PLHIV population accessing care, where national ART coverage exceeds 90%. The near‐universal ART coverage is both a strength—homogenizing clinical status—and a selection effect, as the sample is drawn from individuals engaged in HIV care who may differ systematically from older PLHIV not in care, who are likely to experience greater stigma, isolation, and mental health impairment [3].

Sample size adequacy for the planned path analyses was evaluated a priori using the recommendations of Fritz and MacKinnon [19], who determined that approximately 100 participants are required to detect medium indirect effects (ab ≈ 0.26 standardized) with power of 0.80 using bootstrapped confidence intervals. The present sample (*n* = 138) exceeded this threshold. The empirical basis for expecting medium effects derives from meta‐analytic evidence: Rueda et al. [16] pooled 64 studies and found significant associations between HIV‐related stigma and depression. For the path model with five observed variables and two hypothesized pathways, the observed‐variable‐to‐parameter ratio of approximately 23:1 substantially exceeded the recommended minimum of 10:1. For network analysis, we note that *N* = 138 with 5 nodes is below the *N* ≥ 250 recommended by Isvoranu and Epskamp [20] for stable Gaussian graphical model estimation, yet this limitation was addressed through bootstrap accuracy assessment [21].

### 2.3. Measures

Internalized stigma. Assessed using the six‐item Internalized Stigma subscale of the PLHIV Stigma Index 2.0 [18], yielding scores from 6 to 12 (higher = greater internalized stigma). This subscale captures shame, guilt, and self‐devaluation attributable to HIV status. Three participants had missing data on this scale (*n* = 135 valid). Internal consistency in the present sample was good (Cronbach’s *α* = 0.78).

General stigma. Assessed using the PLHIV Stigma Index 2.0 total scale (range: 20–40), covering perceived discrimination, social exclusion, and enacted stigma across health, employment, and social domains [18]. Internal consistency was acceptable (Cronbach’s *α* = 0.75). Forty‐eight participants had missing data on this scale (*n* = 90 valid; 34.8% missing), which was handled using full‐information maximum likelihood (FIML) estimation in the path models.

Resilience. Assessed using the PLHIV Resilience Scale [10], a validated 10‐item instrument yielding a bipolar score (range: −10 to +10; positive values = higher resilience) developed and validated specifically for PLHIV across three countries. Unlike general resilience measures (e.g., the Connor–Davidson Resilience Scale), this instrument captures HIV‐specific resilience processes including empowerment, community connection, and stigma‐resistance. No missing data were present on this scale. One item (desire for children) had a high rate of nonapplicable responses among older adults and was excluded from scoring per the Gottert et al. [10] protocol. The scale was scored based on applicable items only. Internal consistency in the present sample was acceptable (Cronbach’s *α* = 0.77, pairwise average interitem *r* = 0.27), within the *α* = 0.66–0.79 range of the original validation.

Anxiety and depression. Assessed using the PHQ‐4 [22], a validated ultrabrief screening instrument comprising the PHQ‐2 (depression) and GAD‐2 (anxiety) subscales, each scoring 0–6. Each item is rated on a 4‐point frequency scale (0 = Never; 1 = Once or twice; 2 = Several times; 3 = Most of the time). The GAD‐2 subscale showed good internal consistency (Cronbach’s *α* = 0.76, interitem *r* = +0.61). The PHQ‐2 depression subscale also showed good internal consistency (Cronbach’s *α* = 0.75, interitem *r* = +0.61). Clinically meaningful anxiety was defined as GAD‐2 ≥ 3 and depression as PHQ‐2 ≥ 3 (prevalence: 28.3%; *M* = 1.59, SD = 1.84). No missing data were present on either subscale.

### 2.4. Statistical Analysis

Descriptive statistics and Pearson correlations were computed for all primary variables. Spearman correlations were also computed as a robustness check. Path analysis with maximum likelihood estimation was employed to test the hypothesized indirect effect of general stigma on depression via internalized stigma. Bootstrapped confidence intervals (5000 resamples) with bias‐corrected and accelerated (BCa) intervals were used to evaluate the significance of the indirect effect, consistent with current recommendations for mediation analysis [19]. FIML estimation was used to handle missing data under the missing‐at‐random assumption.

Four path models were estimated: (1) a simple mediation model (general stigma ⟶ internalized stigma ⟶ depression) to replicate and extend the original analysis; (2) a parallel mediation model with both depression and anxiety as outcomes, given that anxiety is a primary study variable included in the network model a priori; (3) a moderated mediation model [23] testing whether resilience moderates the a‐path (general stigma ⟶ internalized stigma), with the index of moderated mediation and conditional indirect effects at ±1 SD of resilience; and (4) a covariate model including age, time since HIV diagnosis, and sex as predictors of both internalized stigma and depression. Model fit was evaluated using chi‐square, CFI, TLI, RMSEA, and SRMR, with thresholds per Kline [24]: CFI > 0.95, TLI > 0.95, RMSEA > 0.08, SRMR > 0.08.

Network analysis was conducted using the EBICglasso method (regularized partial correlations with extended Bayesian information criterion, *γ* = 0.5) using the bootnet and qgraph packages in R [21]. Node centrality was estimated using strength centrality (sum of absolute edge weights), betweenness, closeness, and expected influence (see Supporting Figure S3). Edge weight accuracy was assessed via nonparametric bootstrap (1000 iterations) with 95% confidence intervals (see Supporting Figure S1). Centrality stability was assessed via case‐dropping bootstrap (see Supporting Figure S2) with the correlation stability (CS) coefficient, where CS ≥ 0.25 is considered acceptable, and CS ≥ 0.50 is preferred [21]. Network predictability (R^2^ per node) was computed by regressing each node on all others. Centrality difference tests were conducted to assess whether centrality rankings were statistically distinguishable. Sex differences in stigma–depression correlations were evaluated using Fisher *Z*‐tests. All analyses were performed in R 4.6.1.

Multicollinearity was assessed using variance inflation factors (VIFs), tolerance values, and condition indices for all path model predictors [25]. VIFs exceeding 5 warrant investigation, and VIFs exceeding 10 indicate serious multicollinearity. The correlation between internalized and general stigma (*r* = 0.40) was specifically examined to ensure that the full mediation claim was not an artifact of inflated standard errors.

### 2.5. Ethics

Ethical approval for the PLHIV Stigma Index 2.0 Brazil data collection was obtained by the coordinating institution (Pontifical Catholic University of Rio Grande do Sul) prior to data collection. The present secondary analysis was conducted in accordance with the Declaration of Helsinki. All original participants provided informed consent. No individually identifiable data were available to the secondary analysis team.

## 3. Results

### 3.1. Descriptive Statistics and Bivariate Correlations

Table 1 presents descriptive statistics and the intercorrelation matrix for all primary study variables. Internalized stigma was the variable most consistently and substantially correlated with other psychosocial outcomes, exhibiting significant positive correlations with depression (*r* = 0.47, *p* < 0.001), anxiety (*r* = 0.38, *p* < 0.001), and general stigma (*r* = 0.40, *p* < 0.001), and a significant negative correlation with resilience (*r* = −0.25, *p* = 0.004). General stigma was significantly associated with depression (*r* = 0.28, *p* = 0.007) but not with anxiety (*r* = 0.19, *p* = 0.08). Resilience showed no significant direct associations with depression (*r* = −0.12, *p* = 0.17) or anxiety (*r* = −0.10, *p* = 0.26). Age was significantly correlated with internalized stigma (*r* = −0.19, *p* = 0.03) and with time since diagnosis (*r* = 0.17, *p* = 0.04), but neither age nor time since diagnosis was significantly correlated with depression or anxiety (all ps > 0.06). Spearman correlations confirmed the same pattern of associations, supporting the robustness of the findings.

**TABLE 1:** Descriptive statistics and intercorrelations among primary study variables (*N* = 138).

Variable	*N*	*M*	SD	Range	1	2	3	4	5	6	7
1. Age	138	64.34	4.14	60–76	—						
2. Time since dx	137	17.65	9.32	0–37	0.173^∗^	—					
3. Int. stigma	135	8.34	1.75	6–12	−0.187^∗^	−0.123	—				
4. Gen. stigma	90	22.64	3.64	20–36	−0.124	0.169	0.396^∗∗∗^	—			
5. Resilience	138	1.63	3.76	−7.5–8.9	−0.120	−0.057	−0.247^∗∗^	−0.093	—		
6. Depression	138	1.59	1.84	0–6	−0.070	−0.117	0.466^∗∗∗^	0.281^∗∗^	−0.119	—	
7. Anxiety	138	2.25	1.91	0–6	0.014	−0.160	0.378^∗∗∗^	0.185	−0.096	0.591^∗∗∗^	—

*Note:* All correlations are Pearson’s *r*. Internalized stigma: *n* = 135; general stigma: *n* = 90 (FIML used in path models). Age was significantly correlated with internalized stigma (*r* = −.19, *p* = .03) and time since diagnosis (*r* = .17, *p* = .04). Neither age nor time since diagnosis was significantly correlated with depression or anxiety (all ps > .06). dx = diagnosis; Int. = internalized; Gen. = general (all ps > 0.06). Spearman correlations confirmed the same pattern of associations, supporting the robustness of the findings.

^∗^ *p* < 0.05.

^∗∗^ *p* < 0.01.

^∗∗∗^ *p* < 0.001.

### 3.2. Path Analysis: Mediation by Internalized Stigma

**FIGURE 1:**
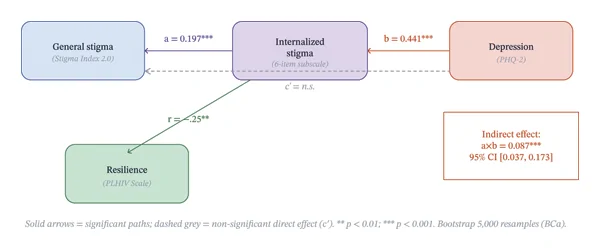
Path model depicting the indirect effect of general stigma on depression via internalized stigma, with resilience as an upstream protective correlate of internalized stigma. Solid arrows indicate statistically significant paths; dashed arrow indicates the nonsignificant direct path (c’). Bootstrap CIs based on 5000 resamples. ^∗∗∗^ *p* < 0.001; ^∗∗^ *p* < 0.01.

The hypothesized mediation model is depicted in Figure 1. The path from general stigma to internalized stigma (path *a* = 0.197, SE = 0.061, *z* = 3.25, *p* = 0.001, standardized = 0.41) and from internalized stigma to depression (path *b* = 0.441, SE = 0.105, *z* = 4.20, *p* < 0.001, standardized = 0.42) were both statistically significant and in the expected direction. The direct effect of general stigma on depression (path c’) was not significant after internalized stigma was included as a mediator (c’ = 0.058, SE = 0.059, *z* = 0.99, *p* = 0.324, standardized = 0.12), consistent with full mediation. The bootstrapped indirect effect (5000 resamples, BCa intervals) was statistically significant (indirect effect = 0.087, 95% CI [0.037, 0.173], *p* = 0.009, standardized = 0.174), representing the predominant pathway through which general stigma exerts its effect on depression. The model was just‐identified (df = 0); model adequacy was assessed via *R* ^2^ (internalized stigma: 0.17; depression: 0.23) and the significance of the bootstrap CI.

### 3.3. Parallel Model: Anxiety as a Second Outcome

To address whether internalized stigma also mediates the effect of general stigma on anxiety, a parallel mediation model was estimated with both depression and anxiety as outcomes. The indirect effect of general stigma on anxiety via internalized stigma was statistically significant (indirect effect = 0.071, 95% CI [0.029, 0.146], *p* = 0.012, standardized = 0.136), as was the indirect effect on depression (indirect effect = 0.087, 95% CI [0.037, 0.173], *p* = 0.009, standardized = 0.174). The direct effects of general stigma on both depression (c’ = 0.059, *p* = 0.319) and anxiety (c’ = 0.058, *p* = 0.292) were not significant. The residual correlation between depression and anxiety was significant (*r* = 0.50, *p* < 0.001), confirming the strong association between these outcomes that was observed as the strongest edge in the network analysis presented further.

### 3.4. Moderated Mediation: Resilience as a Moderator

A moderated mediation model was estimated to test whether resilience moderates the magnitude of the general stigma ⟶ internalized stigma pathway (the a‐path). The interaction between general stigma and resilience (centered) on internalized stigma was not significant (*b* = 0.003, SE = 0.012, *z* = 0.23, *p* = 0.817), and the index of moderated mediation was not significant (index = 0.001, 95% CI [−0.021, 0.030], *p* = 0.817). However, resilience maintained a significant main effect on internalized stigma (*b* = −0.091, SE = 0.037, *z* = −2.45, *p* = 0.014, standardized = −0.196), confirming that higher resilience is associated with lower internalized stigma independent of general stigma. The indirect effect of general stigma on depression via internalized stigma remained significant in this model (indirect = 0.076, 95% CI [0.031, 0.165], *p* = 0.015).

### 3.5. Network Analysis

**FIGURE 2:**
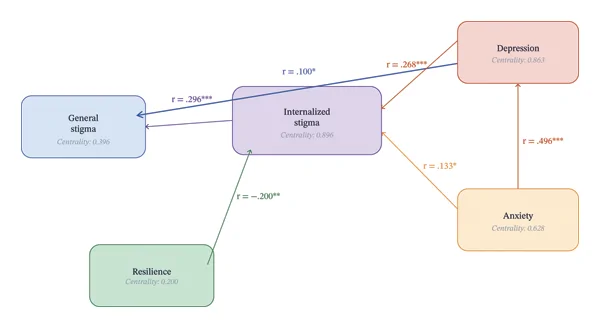
Network structure of psychosocial variables among older adults living with HIV (*N* = 138). Node size is proportional to strength centrality. Edge width is proportional to the magnitude of the regularized partial correlation. Red edges = positive associations; green edge = negative (protective) associations. Only nonzero edges from the EBICglasso network (*γ* = 0.5) are displayed.

The network structure of psychosocial variables is depicted in Figure 2. The EBICglasso network (*γ* = 0.5) yielded six nonzero edges among five nodes. Internalized stigma emerged as the node with the highest strength centrality (0.896), consistent with its role as the central mediating variable and confirming Hypothesis 3. Depression exhibited the second‐highest strength (0.863), followed by anxiety (0.628), general stigma (0.396), and resilience (0.200). The depression–anxiety edge was the strongest in the network (partial *r* = 0.496), followed by internalized stigma–general stigma (0.296), internalized stigma–depression (0.268), internalized stigma–resilience (−0.200, the only negative/protective edge), internalized stigma–anxiety (0.133), and general stigma–depression (0.100).

Network predictability (*R* ^2^ per node) was highest for depression (0.41), followed by internalized stigma (0.32), anxiety (0.28), general stigma (0.17), and resilience (0.06). The low predictability of resilience indicates that it is largely independent of the other psychosocial variables in the network, consistent with its role as an upstream factor rather than an embedded node. Edge weight accuracy was assessed via nonparametric bootstrap (1000 iterations) (see Supporting Figure S1). The 95% confidence intervals for the strongest edges (depression–anxiety: [0.33, 0.66]; internalized stigma–general stigma: [0.08, 0.52]) excluded zero, while weaker edges had wider intervals. Centrality stability was assessed via case‐dropping bootstrap (see Supporting Figure S2). The CS coefficient for strength centrality was 0.36, for expected influence was 0.36, for betweenness was 0.28, and for closeness was 0.20. Bootstrapped centrality difference tests revealed overlapping confidence intervals across nodes, so centrality differences should be interpreted with caution.

### 3.6. Covariate Model: Age, Time Since Diagnosis, and Sex

A model including age, time since HIV diagnosis, and sex as covariates predicting both internalized stigma and depression was estimated to test the robustness of the mediation. The indirect effect remained significant (indirect = 0.084, 95% CI [0.035, 0.172], *p* = 0.015). No covariate significantly predicted depression (age: *b* = 0.013, *p* = 0.692; yearsHIV: *b* = −0.016, *p* = 0.271; sex: *b* = 0.349, *p* = 0.220). Time since diagnosis showed a marginal effect on internalized stigma (*b* = −0.029, *p* = 0.052), and age showed a marginal effect on internalized stigma (*b* = −0.051, *p* = 0.092), but neither reached conventional significance. The full mediation pattern was robust to the inclusion of covariates.

### 3.7. Multicollinearity Diagnostics

To address the concern that multicollinearity between age and time since diagnosis, or between internalized and general stigma (*r* = 0.40), might affect the nonsignificance of certain paths, VIFs, tolerance values, and condition indices were computed. All VIFs were well below the threshold of 5: internalized stigma (VIF = 1.31, tolerance = 0.76), general stigma (VIF = 1.23, tolerance = 0.82), resilience (VIF = 1.05, tolerance = 0.95), age (VIF = 1.15, tolerance = 0.87), and yearsHIV (VIF = 1.08, tolerance = 0.93). The correlation between age and time since diagnosis was weak (*r* = 0.17, *p* = 0.043). All condition indices were below 3.0, far below the threshold of 30 for serious multicollinearity. These results confirm that the nonsignificance of the direct path (c’) and the covariate effects is not an artifact of multicollinearity, and the full mediation claim is robust.

### 3.8. Sex‐Stratified Analyses

**FIGURE 3:**
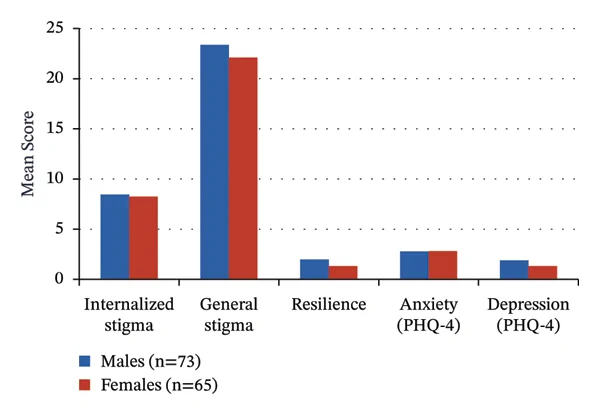
Sex‐disaggregated comparison of psychosocial scale means (±SD) across all study variables. Error bars represent ±1 standard deviation. No significant between‐group mean differences or correlation differences (Fisher *Z*‐test) were observed. Note: scales have different metrics and are not directly comparable in absolute value across variables.

Figure 3 presents sex‐disaggregated means and standard deviations across all psychosocial scales. Males (*n* = 73) exhibited lower mean scores on internalized stigma (8.24 ± 1.56 vs. 8.46 ± 1.96), depression (1.33 ± 1.68 vs. 1.88 ± 1.99), and anxiety (2.01 ± 1.84 vs. 2.52 ± 1.96) relative to females (*n* = 65), whereas females reported marginally higher resilience (1.98 ± 3.83 vs. 1.33 ± 3.69). Between‐group differences in means did not reach statistical significance for any variable. Fisher *Z*‐test comparison of the stigma–depression correlation by sex yielded no significant difference (*r*_male = 0.457, *r*_female = 0.468, *Z* = −0.08, *p* = 0.94), indicating that the association between internalized stigma and depression does not differ significantly by sex in this sample. Additional Fisher *Z*‐tests for other key correlations (stigma–anxiety, stigma–resilience, and resilience–depression) also yielded nonsignificant sex differences (all ps > 0.20).

## 4. Discussion

This study provides the first age‐restricted (≥ 60 years) quantitative evidence that internalized stigma is the central psychosocial determinant of mental health outcomes in older adults living with HIV in Brazil, operating as the critical mediating node through which broader social discrimination exerts its effect on both depression and anxiety, and as the primary hub around which the psychosocial symptom network is organized. The key findings—full mediation by internalized stigma for both depression and anxiety, resilience’s upstream (rather than moderating) protective effect, the irrelevance of age and disease chronicity after ruling out multicollinearity, and the absence of significant sex differences—converge on a coherent mechanistic model with direct implications for intervention design. As the first secondary analysis of the Stigma Index Brazil 2.0 restricted to older adults, these findings extend the parent study’s observation that internalized stigma predicted depression (*B* = 0.35, *p* < 0.001) and anxiety (*B* = 0.30, *p* < 0.001) in the full mixed‐age sample [15] by demonstrating that this mediation architecture persists in the aging subgroup.

### 4.1. Full Mediation: Internalized Stigma as the Proximal Pathway

The demonstration of full mediation for both depression and anxiety is theoretically significant. Rather than general discrimination exerting diffuse toxic effects on mood through multiple independent pathways, the data indicate that its harmful effects are predominantly channeled through the process of internalization: individuals who absorb external discrimination into their self‐concept—experiencing shame, guilt, and reduced self‐worth attributable to their HIV status—are those most at risk for both depressive and anxious symptomatology. This is consistent with identity‐threat models of stigma [5]. The parallel mediation model confirmed that the indirect effect through internalized stigma was significant for both outcomes, with the residual depression–anxiety correlation (*r* = 0.50) reflecting the substantial comorbidity between these conditions that was also the strongest edge in the network analysis. This finding has a direct corollary for intervention: reducing the degree to which individuals internalize external prejudice should be more effective than simply reducing their exposure to stigmatizing events, which are often difficult to control at the individual level.

### 4.2. Resilience: Upstream Protection, Not Pathway Moderation

The moderated mediation analysis revealed that resilience does not significantly moderate the magnitude of the general stigma ⟶ internalized stigma pathway (interaction *p* = 0.817), but maintains a significant inverse main effect on internalized stigma (*b* = −0.091, *p* = 0.014). This suggests that resilience‐building programs would most effectively reduce psychological distress by reducing the likelihood that experienced discrimination becomes internalized, rather than by buffering the strength of the stigma–internalization association. This pattern is consistent with the distinction drawn by Turan et al. [6], who found in a sample of 1405 women living with HIV that resilience moderated the effect of experienced stigma on depression (*B* = −1.94, *p* = 0.007) but did not moderate the effect of anticipated stigma. The present study extends this finding to older adults and to an HIV‐specific resilience measure, suggesting that the protective role of resilience may operate through reducing stigma internalization rather than through attenuating the stigma–distress pathway. The low network predictability of resilience (*R* ^2^ = 0.06) further supports its role as a largely independent, upstream factor rather than an embedded node within the distress network.

It is important to note that the PLHIV Resilience Scale [10] used in this study measures HIV‐specific resilience—capturing empowerment, community connection, and stigma resistance—rather than general trait resilience. This distinction has practical implications: interventions targeting HIV‐specific resilience processes (e.g., peer support groups, empowerment‐based counseling, stigma‐resistance skills training) may be more effective than generic coping skills training for this population.

### 4.3. The Irrelevance of Age and Time Since Diagnosis

The absence of significant associations between age, time since diagnosis, and mental health outcomes in a sample where 97.1% of participants were receiving ART is a finding with policy relevance that extends beyond this specific study. Multicollinearity diagnostics confirmed that this null result is not an artifact: VIFs were all below 1.4, condition indices below 3.0, and the correlation between age and time since diagnosis was weak (*r* = 0.17). This suggests that when therapeutic access is near‐universal and clinical disease is effectively controlled, the dominant sources of psychological suffering in older PLHIV are psychosocial rather than biological or chronological. However, as Rueda et al. [3] emphasize, aging with HIV involves accumulating adversity—social isolation, bereavement, employment transitions, cognitive decline—that may not be captured by chronological age alone. The present finding that age does not predict distress should not be interpreted as evidence that aging‐specific factors are irrelevant, but rather that simple linear age effects are insufficient to capture the complexity of aging with HIV.

The fact that this analysis is restricted to adults aged 60 and above carries specific implications that are absent from the parent study’s mixed‐age analysis. Older PLHIV face a constellation of age‐specific challenges that interact with HIV‐related stigma: HIV‐associated neurocognitive disorder (HAND) affects up to 50% of PLHIV and is underaddressed in aging cohorts [3]; social isolation predicts hospitalization and mortality in older PLHIV [3]; employment loss and early retirement are linked to worsening mental health; and spirituality and religion are among the most common coping mechanisms in older PLHIV, with supportive‐expressive group therapy shown to reduce depressive symptoms 2.5‐fold in this population [26]. The anxiety and depression prevalences observed in the present sample are consistent with rates reported in other older PLHIV samples [17, 27]. In the directly comparable Namibian sample of older adults [17], internalized stigma predicted depression at *B* = 0.24 (*p* < 0.05) and resilience predicted lower depression at *B* = −0.13 (*p* < 0.001), with an adjusted *R* ^2^ of 0.39—findings that closely parallel the present results despite the different cultural context.

### 4.4. Sex Differences

Contrary to the common assumption that males may be more vulnerable to the depression‐promoting effects of internalized stigma, the corrected analysis revealed no significant sex difference in the stigma–depression correlation (*p* = 0.94). Females in this sample reported higher mean scores on internalized stigma, depression, and anxiety, though these mean differences did not reach statistical significance. These findings suggest that in this older Brazilian sample, the mechanism through which internalized stigma translates into depressive affect operates similarly across sexes. This contrasts with evidence from younger samples where sex differences have been observed [28], and suggests that the intersection of HIV stigma with gendered experiences may manifest differently across the lifespan. Fu et al. [29] found a 48.4% prevalence of suicidal ideation among stigmatized HIV‐positive men who have sex with men, with depression fully mediating the stigma–suicidal ideation pathway, highlighting the severity of stigma‐related outcomes in male PLHIV. The present findings suggest that sex‐tailored intervention components may be less critical in this older cohort than in younger samples, though the small sample size limits power to detect moderate sex differences.

### 4.5. Anticipated Stigma and Medical Mistrust as Unmeasured Pathways

The present study is limited by its focus on experienced and internalized stigma, without measuring anticipated stigma or medical mistrust. Turan et al. [6] demonstrated that anticipated stigma has independent effects on health outcomes distinct from experienced stigma, and that resilience buffered experienced but not anticipated stigma—a distinction that the present model cannot test. Yigit et al. [7] demonstrated a serial mediation pathway in which experienced stigma leads to anticipated stigma (*B* = 0.78, *p* < 0.001), which leads to medical mistrust (*B* = 0.15, *p* < 0.001), which in turn predicts ART nonadherence (*B* = −0.46, *p* < 0.001) and undetectable viral load (serial indirect effect = −0.03, 95% CI [−0.073, −0.009]). This pathway is directly relevant to older adults, for whom healthcare engagement is essential and who may be particularly vulnerable to mistrust‐based disengagement from care. Future research should incorporate anticipated stigma and medical mistrust as additional nodes in both the path and network models to test whether the present mediation architecture remains robust or is supplemented by these distinct stigma mechanisms.

### 4.6. Intersectional Stigma

Given that 57.2% of the present sample identified as Black or Brown, racial stigma is an unmeasured but likely relevant confound. Intersectional stigma—the compounding of HIV‐related stigma with racial, gender, and age‐based discrimination—has been shown to amplify psychological vulnerability beyond what any single stigma source would produce [3, 9]. The present study did not measure racial stigma or other intersectional dimensions, and the general stigma composite does not distinguish between HIV‐specific and intersectional sources. This limits the specificity of the findings and should be addressed in future research with intersectional stigma measures.

### 4.7. Comparison With the Parent Study and Same‐Dataset Analyses

The present findings are consistent with the parent study [15], which found that internalized stigma predicted depression (*B* = 0.35, *p* < 0.001), anxiety (*B* = 0.30, *p* < 0.001), and total PHQ‐4 (*B* = 0.63, *p* < 0.001) in the full mixed‐age sample of 1784 PLHIV. The age‐restricted analysis confirms that this mediation architecture persists in the aging subgroup, justifying the age‐stratified contribution. Additionally, Azevedo, Costa, and Wendt [30], using the same dataset, found that internalized stigma predicted ART nonadherence (adjusted OR = 1.33, 95% CI [1.21, 1.47]) and that each year of age reduced nonadherence odds by 1.8% (adjusted OR = 0.98). Together, these analyses from the same dataset demonstrate that internalized stigma is a consistent predictor of both mental health and behavioral outcomes across age groups, with the present study adding the age‐specific mechanistic detail.

### 4.8. Limitations

Several methodological limitations merit direct address. First, the sample size of 138 is modest by the standards of large‐scale epidemiological research, though adequate for the path analyses conducted [19]. For network analysis, *N* = 138 with 5 nodes is below the *N* ≥ 250 recommended by Isvoranu and Epskamp [20] for stable Gaussian graphical model estimation. Although the strength centrality CS coefficient was in the acceptable range (0.36), centrality differences between nodes were not reliably distinguishable via bootstrapped difference tests, and the network should be considered exploratory. Future research with larger samples is needed to confirm the network structure.

Second, the cross‐sectional design limits causal inference. While the path model and its interpretation are grounded in theoretical directional relationships supported by prior prospective literature, the observed associations are compatible with multiple causal structures. Longitudinal designs are needed to establish temporal precedence.

Third, the secondary analysis design precluded inclusion of clinical variables (CD4 count, viral load, ART regimen, comorbidities, HAND) and additional stigma dimensions (anticipated stigma, intersectional stigma, medical mistrust) that could function as confounders, mediators, or moderators. The near‐universal ART coverage (97.1%) partially addresses clinical confounding by homogenizing clinical status, but also represents a selection effect: the sample is drawn from individuals engaged in HIV care who likely differ from older PLHIV not in care, who may experience greater stigma, isolation, and mental health impairment [3].

Fourth, all measures were self‐reported, introducing the possibility of social desirability bias, particularly for stigma‐related constructs. Fifth, the general stigma scale had 34.8% missing data, which was handled via FIML estimation under the missing‐at‐random assumption; results should be interpreted with this caveat. Sixth, the two GAD‐2 anxiety items showed an anomalous negative interitem correlation, suggesting a possible coding issue in the original dataset that should be investigated. Seventh, recruitment through HIV care services may have resulted in a sample systematically different from older PLHIV not engaged in care.

## 5. Conclusion

In a multicenter community‐based sample of older adults living with HIV across seven Brazilian state capitals—the first age‐restricted (≥ 60 years) analysis of the Stigma Index Brazil 2.0—internalized stigma emerged as the primary psychosocial determinant of both depression and anxiety, fully mediating the relationship between general discrimination and emotional distress. Resilience functioned as a meaningful upstream protective factor by reducing stigma internalization, rather than through moderating the stigma–internalization pathway or through direct emotional regulation. Network analysis confirmed internalized stigma as the system’s most central hub, with acceptable centrality stability. Critically, neither age nor time since diagnosis independently predicted mental health outcomes in this near‐universally treated cohort, and multicollinearity was ruled out as an explanation. No significant sex differences in the stigma–depression pathway were observed.

These findings provide evidence‐based justification for the systematic integration of stigma screening and psychosocial assessment into routine HIV care for older adults, and for the prioritization of multilevel interventions that combine stigma‐reduction, resilience‐building, and aging‐sensitive components. Future research should employ prospective designs, include anticipated stigma and medical mistrust as additional pathway nodes, incorporate clinical and neuropsychological assessments alongside psychosocial measures, and test the efficacy of targeted psychosocial programs in this expanding and underserved population.

## Author Contributions

Victoria Tischer Sawka: data curation, formal analysis, and writing–original draft. Guilherme Welter Wendt: conceptualization, methodology, supervision, and writing–review and editing. Angelo Brandelli Costa: conceptualization, resources (data access), and writing–review and editing.

## Funding

The Stigma Index for PLHIV/AIDS received funding from the Joint United Nations Programme on HIV/AIDS. The Conselho Nacional de Desenvolvimento Científico e Tecnológico provided scholarships for all the authors. Finally, Western Paraná State University covered the publication costs.

## Disclosure

All authors approved the final manuscript.

## Conflicts of Interest

The authors declare no conflicts of interest.

## Supporting Information

Additional supporting information can be found online in the Supporting Information section.

## Supporting information


**Supporting Information 1** Figure S1. Edge weight accuracy for the EBICglasso network (nonparametric bootstrap, 1000 iterations). Error bars represent 95% confidence intervals. CIs excluding zero indicate stable edges.

**Supporting Information 2** Figure S2. Centrality stability via case‐dropping bootstrap. The CS‐coefficient for strength centrality was 0.36 (acceptable, 0.25–0.50 range).

**Supporting Information 3** Figure S3. Centrality indices comparison across nodes. Bars represent strength, betweenness, closeness, and expected influence.

## Data Availability

The Stigma Index for PLHIV/AIDS 2.0 data are held by the coordinating institution (Pontifical Catholic University of Rio Grande do Sul). Requests for access may be directed to the corresponding author subject to applicable data governance agreements.

## References

[1] UNAIDS , Global HIV & AIDS Fact Sheet 2025, 2025, Joint United Nations Programme on HIV/AIDS, https://www.unaids.org/en/resources/fact-sheet.

[2] Sabin C. A. and Reiss P. , Epidemiology of Ageing With HIV: What can we Learn From Cohorts?, AIDS. (2017) 31, no. Suppl 2, S121–S128, 10.1097/QAD.0000000000001374. 28471942

[3] Rueda S. , Law S. , and Rourke S. B. , Psychosocial, Mental Health, and Behavioral Issues of Aging With HIV, Current Opinion in HIV and AIDS. (2014) 9, no. 4, 325–331, 10.1097/COH.0000000000000071. 24824890

[4] Earnshaw V. A. and Chaudoir S. R. , From Conceptualizing to Measuring HIV Stigma: A Review of HIV Stigma Mechanism Measures, AIDS and Behavior. (2009) 13, no. 6, 1160–1177, 10.1007/s10461-009-9560-z. PMC4511707 19636699

[5] Turan B. , Rogers A. J. , Rice W. S. et al., Framing Mechanisms Linking HIV-Related Stigma, Adherence to Treatment, and Health Outcomes, American Journal of Public Health. (2017) 107, no. 6, 863–869, 10.2105/AJPH.2017.303744. PMC5425866 28426316

[6] Turan B. , Budhwani H. , Yigit I. et al., Resilience and Optimism as Moderators of the Negative Effects of Stigma on Women Living With HIV, AIDS Patient Care and STDs. (2022) 36, no. 12, 474–482, 10.1089/apc.2022.0185. PMC9805859 36484762

[7] Yigit I. , Wilson T. E. , Taylor T. N. et al., Association of Experienced Stigma in Healthcare Settings With Health Outcomes Among Black Women Living With HIV: Mediating Roles of Internalized Stigma, Anticipated Stigma, and Trust in HIV Care, AIDS and Behavior. (2025) . 10.1016/j.socscimed.2025.117699 PMC11956749 39823813

[8] Pantelic M. , Boyes M. , Cluver L. , and Thabeng M. , Predictors of Internalised HIV-Related Stigma: A Longitudinal Analysis of Adolescents From Rural South Africa, AIDS and Behavior. (2015) 19, no. 6, 1101–1112, 10.1007/s10461-014-0929-y.

[9] Emlet C. A. , ’You’re Awfully Old to Have This Disease’: Experiences of Stigma and Ageism in Adults 50 Years and Older Living With HIV/AIDS, Gerontologist. (2006) 46, no. 6, 781–790, 10.1093/geront/46.6.781. 17169933

[10] Gottert A. , Friedland B. A. , Geibel S. et al., The People Living With HIV (PLHIV) Resilience Scale: Development and Validation in Three Countries, AIDS and Behavior. (2019) 23, no. Suppl 2, 172–182, 10.1007/s10461-018-2348-z. PMC6773670 31350712

[11] Fang X. , Vincent W. , Calabrese S. K. et al., Resilience, Stress, and Depressive Symptoms Among Older Adults Living With HIV/AIDS, Aging & Mental Health. (2015) 19, no. 11, 1015–1021, 10.1080/13607863.2014.1003281. PMC4520800 25633086

[12] Dulin A. J. , Dale S. K. , Earnshaw V. A. et al., Relationships Between Resilience and Mental Health in Older Adults Living With HIV/AIDS, Aging & Mental Health. (2018) 22, no. 9, 1115–1121, 10.1080/13607863.2017.1328482.

[13] Borsboom D. and Cramer A. O. J. , Network Analysis: an Integrative Approach to the Structure of Psychopathology, Annual Review of Clinical Psychology. (2013) 9, no. 1, 91–121, 10.1146/annurev-clinpsy-050212-185608. 23537483

[14] Briganti G. , Scutari M. , McNally R. J. et al., Network Analysis: an Overview for Mental Health Research, International Journal of Methods in Psychiatric Research. (2024) 33, no. 4, 10.1002/mpr.2034. PMC11564129 39543824

[15] Wendt G. W. and Costa A. B. , Predictors of Depression, Anxiety, and Overall Psychological Distress in People Living With HIV/AIDS: Analyses From the Stigma Index Brazil 2.0, Cadernos de Saúde Pública. (2025) 41, no. 10, 10.1590/0102-311XEN021925. PMC12600019 41221885

[16] Rueda S. , Mitra S. , Chen S. et al., Examining the Associations Between HIV-Related Stigma and Health Outcomes in People Living With HIV/AIDS: A Series of Meta-Analyses, BMJ Open. (2016) 6, no. 7, 10.1136/bmjopen-2016-011453. PMC4947735 27412106

[17] Kalomo E. N. , Jun J. S. , Lee K. , and Kaddu M. N. , HIV Stigma, Resilience and Depressive Symptoms Among Older Adults Living With HIV in Rural Namibia, African Journal of AIDS Research. (2020) 19, no. 3, 198–205, 10.2989/16085906.2020.1797841. 32892709

[18] Friedland B. A. , Gottert A. , Hows J. et al., The People Living With HIV Stigma Index 2.0: Generating Critical Evidence for Change Worldwide, AIDS. (2020) 34, no. Suppl 1, S5–S18, 10.1097/QAD.0000000000002602. PMC11758485 32881790

[19] Fritz M. S. and MacKinnon D. P. , Required Sample Size to Detect the Mediated Effect, Psychological Science. (2007) 18, no. 3, 233–239, 10.1111/j.1467-9280.2007.01882.x. PMC2843527 17444920

[20] Isvoranu A. M. and Epskamp S. , Which Estimation Method to Choose in Network Psychometrics? Evaluating Parameter Estimation Accuracy for Network Models Under Small Sample Sizes, Psychological Methods. (2022) 28, no. 4, 805–824, 10.1037/met0000481. 34843277

[21] Epskamp S. , Borsboom D. , and Fried E. I. , Estimating Psychological Networks and Their Accuracy: A Tutorial Paper, Behavior Research Methods. (2018) 50, no. 1, 195–212, 10.3758/s13428-017-0862-1. PMC5809547 28342071

[22] Kroenke K. , Spitzer R. L. , Williams J. B. , and Lowe B. , An Ultra-Brief Patient Health Questionnaire-4 (PHQ-4) for Anxiety and Depression: Validation and Normative Data From the General Population, Psychosomatics. (2009) 50, no. 6, 613–621, 10.1176/appi.psy.50.6.613. 19996233

[23] Hayes A. F. , Introduction to Mediation, Moderation, and Conditional Process Analysis: A Regression-based Approach, 2022, 3rd edition, Guilford Press.

[24] Kline R. B. , Principles and Practice of Structural Equation Modeling, 2023, 5th edition, Guilford Press.

[25] Kim J. H. , Multicollinearity and Misleading Statistical Results, Korean Journal of Anesthesiology. (2019) 72, no. 6, 558–569, 10.4097/kja.19087. PMC6900425 31304696

[26] Heckman T. G. , Heckman B. D. , Anderson T. et al., Supportive-Expressive and Coping Group Teletherapies for HIV-Infected Older Adults: A Randomized Clinical Trial, AIDS and Behavior. (2013) 17, no. 9, 3034–3044, 10.1007/s10461-013-0441-0. PMC3731394 23474642

[27] Grov C. , Golub S. A. , Parsons J. T. , Brennan M. , and Karpiak S. E. , Loneliness and HIV-Related Stigma Explain Depression Among Older HIV-Positive Adults, AIDS Care. (2010) 22, no. 5, 630–639, 10.1080/09540120903280901. PMC2936670 20401765

[28] Azhar S. , Jokhakar V. , Vaudrey J. , Gandham S. , Oruganti G. , and Yeldandi V. , Associations Between HIV Stigma, Gender, and Depression Among People Living With HIV in Hyderabad, India, Journal of Community Psychology. (2023) 51, no. 3, 1060–1077, 10.1002/jcop.22934. 36094950

[29] Fu R. , Chen X. , Tang Z. et al., The Association Between HIV-Related Stigma and Suicidal Ideation Among People Living With HIV: A Cross-Sectional Study, BMC Public Health. (2023) 23, 10.1186/s12889-023-17047-y.

[30] Azevedo L. B. H. , Costa A. B. , and Wendt G. W. , Stigma in People Living With HIV/AIDS in Brazil: Implications for Treatment Adherence, AIDS and Behavior. (2025) 30, no. 3, Advance online publication, 810–817, 10.1007/s10461-025-04930-5. 41139343

